# Availability of public outlets and regular consumption of fruits and vegetables among adolescents in public schools

**DOI:** 10.1590/1984-0462/2022/40/2021062IN

**Published:** 2022-06-10

**Authors:** Aline Daniela da Cruz e Silva, Christiane Opuszka Machado, Aichely Rodrigues da Silva, Doroteia Aparecida Höfelmann

**Affiliations:** a Universidade Federal do Paraná, Curitiba, PR, Brazil.; b Secretaria Municipal de Saúde de Curitiba, Curitiba, PR, Brazil.; c Universidade Federal do Maranhão, Imperatriz, MA, Brazil.

**Keywords:** Food consumption, Spatial distribution, Adolescent, Healthy eating, Food supply, Consumo alimentar, Distribuição espacial, Adolescente, Alimentação saudável, Abastecimento de alimentos

## Abstract

**Objective:**

To assess the association between the presence of public outlets selling fruits and vegetables and the regular intake of these foods by adolescents from public schools in the city of Curitiba, Paraná, Brazil.

**Methods:**

Data collection was carried out by a questionnaire answered by the adolescents. Regular intake was defined as eating fruits and vegetables five or more times a week. Environmental data were obtained by assessing the availability and prices of fruits and vegetables traded in public outlets within a 1.6-km radius from 30 randomly selected public schools.

**Results:**

A total of 1,232 students from 30 public schools participated in the study. 43.4% of the adolescents reported a regular intake of fruits; 67.0% of them reported a regular intake of vegetables. In the schools, fruit intake ranged from 26.8 to 68.0%, and the vegetables intake ranged from 54.8 to 82.2%. A total of 22 schools had fruit and vegetables being traded in their surroundings. Regular intake of vegetables was positively correlated with their variety (r=0.82; p=0.007). The Moran’s local index indicated low fruit intake in a high-supply region; in other three regions with low supply, there was a high intake of fruits; and there was a high consumption of vegetables in a high-supply region.

**Conclusions:**

There are differences in the supply of fruits and vegetables of public outlets in the school’s surroundings as well as in the distribution of regular intake among regions. The density of public outlets and the variety were both associated with greater intake of fruits and vegetables among adolescents of public school.

## RESUMO

**Objetivo:**

Avaliar a associação entre presença de equipamentos públicos de comércio de frutas e hortaliças com seu consumo regular por adolescentes de escolas públicas de Curitiba, Paraná.

**Métodos:**

Informações dos adolescentes foram coletadas por meio de questionário. Considerou-se como regular o consumo de frutas e hortaliças cinco ou mais vezes na semana. Foram incluídos dados do ambiente obtidos por auditagem de equipamentos públicos de venda de frutas e hortaliças no raio de 1,6 km do entorno de 30 escolas estaduais aleatoriamente sorteadas, no qual foram avaliados a disponibilidade e os preços desses alimentos.

**Resultados:**

Participaram do estudo 1.232 alunos de 30 escolas estaduais. O consumo regular de frutas foi referido por 43,4% e de hortaliças por 67,0% dos adolescentes. Nas escolas, o consumo de frutas variou entre 26,8 e 68,0% e o de hortaliças entre 54,8 e 82,2%; 22 escolas contavam com oferta de frutas e hortaliças no entorno. O consumo regular de hortaliças esteve correlacionado positivamente com a sua variedade (r=0,82; p=0,007). O índice de Moran local indicou baixo consumo de frutas em uma regional de alta oferta e, em outras três, alto consumo de frutas e hortaliças para baixa oferta e alto consumo de hortaliças em uma regional de alta oferta.

**Conclusões:**

Existem diferenças na oferta de frutas e hortaliças dos equipamentos públicos no entorno escolar e na distribuição do consumo regular entre as regionais. Características relacionadas à densidade de equipamentos de comércio e à variedade estiveram associadas ao maior consumo de frutas e hortaliças entre os adolescentes de escolas públicas.

## INTRODUCTION

In 2016, in the world, over 340 million children and adolescents aged between 5 and 19 years were overweight.^1^ In Brazil, in 2015, the prevalence of overweight was 23.7%.^2^

Overweight is associated with lower density of places to buy and consume fruits and vegetables, and with greater supply of foods high in sugar and fat. The type of outlet and the availability of food in the neighborhood have a strong influence on the purchase decision of individuals.^3,4^ A study conducted in Florianópolis, state of Santa Catarina, Brazil, identified that shopping in bakeries was associated with excess weight among students of the public network, whereas in the private network shopping in supermarkets was associated with a lower prevalence of overweight.^5^

In Brazil, street markets are characterized by the sale of fresh foods, with greater diversity and easy access, in addition to promoting popular culture, and are an important form of trade of fruits and vegetables.^6^ However, most of the available studies on school environments are concentrated in obesogenic environments such as convenience stores and fast-food restaurants.^7–10^

In adolescence, there is a growing interest and autonomy in the purchase of food, and the presence of food stores in the school environment can influence food choices.^11^ Thus, identifying the supply of fruits and vegetables is important for proposing strategies to increase the consumption of these foods among adolescents. The objective of this study was to evaluate the association between the presence of public outlets for selling fruits and vegetables and the regular consumption of these foods by adolescents from public schools.

## METHOD

This is a cross-sectional study, part of the project entitled *Excesso de peso e características do ambiente escolar de estudantes de Curitiba, Paraná* [“Overweight and characteristics of the school environment of students from Curitiba, Paraná, Brazil”]. The authors used data on students enrolled in state schools from the 6^th^ year of elementary school to the 3^rd^ year of high school, from March 2016 to April 2017, and on the points of sale of fruits and vegetables (root vegetables and greens) that were under the coordination of the Municipal Department of Food Supply of Curitiba (*Secretaria Municipal de Abastecimento de Curitiba* – SMAB) in 2018.

Curitiba, capital of the state of Paraná, is divided into ten administrative regions, aiming to facilitate administration and bring public services closer to the population, in such a way that the composition of the regions guarantees the balance of variables such as the number of urban facilities (schools, healthcare units, etc.) and services provided by municipal agencies, among others.^12^ In 2010, the municipal Human Development Index was 0.823. Nevertheless, the municipality has intra-municipal differences: the average household income (AHI) ranged from BRL 1,674.84 to BRL 6,438.71 among the regions.

To assess the students’ fruit and vegetable consumption, the sample was calculated considering the number of students enrolled from the 6^th^ year of elementary school to the 3^rd^ year of high school in the daytime period of state schools in Curitiba in 2015 (n=110,238), unknown outcome prevalence of 50% (to maximize sample size), margin of error of four percentage points and 95% confidence level, resulting in 597 students. In order to consider the effect of the study design, the sample size was multiplied by two, and the percentage of 20% was added to the total to compensate for refusals, thus totaling 1,437 students. The schools were drawn from the list of state schools, resulting in 30 units. In the administrative regions of the municipality, there was at least one selected school. In each school, one school year and one class were drawn. If the number of students in the class did not reach the number of students for the sample, another class was drawn.

Information on fruit and vegetable consumption, time spent walking to the school, and purchase of food near the school were obtained by applying a questionnaire in the classroom.

The weekly consumption of fruits and vegetables was investigated using a questionnaire on the weekly frequency concerning the consumption of food items, including fruits, cooked vegetables, and raw salad.^13^ The consumption of fruits and vegetables (cooked and raw vegetables) was considered regular when it was equal to or more than five times a week, as described by the National Survey of School Health (*Pesquisa Nacional de Saúde do Escolar* – PENSE, 2015).^14^

As a proxy for the distance between home and school, the question regarding the time perceived by the student when walking to the school (0–10 minutes, 11–20 min, 21–30 min, 31–59 min, one hour or more) was adopted.^14^ The number of days of the week on which the student used to purchase food from the outlet near the school was also investigated.

Data on fruit and vegetable availability were obtained by quantifying the SMAB points of sale of foods. Public outlets were listed according to the SMAB website and delimited on Google Maps for later *in loco* verification of addresses. These outlets consisted of street markets, markets, and fruit stores of free access to the population. Data collection at the public outlets selling fruits and vegetables took place from March to December 2018.

The evaluation of availability and price of fruits and vegetables was performed using the instrument for assessing retail food stores and open-air markets – *ESAO-s Feiras Livres*.^15^ The instrument assesses availability, variety, and the lowest price of 11 fruits (pineapple, banana, orange, apple, papaya, mango, watermelon, tangerine, lime, grape, and melon) and 11 vegetables (lettuce, cabbage, carrot, chayote, cucumber, tomato, onion, zucchini, sweet pepper, eggplant, and green beans) most consumed in Brazil according to the Consumer Expenditure Survey (*Pesquisa de Orçamentos Familiares* – POF 2008–2009). For this research, a fruit (melon) and a vegetable (green beans) with high consumption in the southern region of the country were added.^16^ Availability was obtained by observing the presence of the food on the list (yes; no). Subsequently, the lowest price found in the stands of the street markets was recorded for each item. The register of the lowest price considers that consumers seek to save money when shopping.^15^

Based on the data collected in the audit of the street markets, the Healthy Food Store Index (HFSI) was calculated. The assessment considers availability, variety, and advertising of fruits and vegetables, availability of “fixed price” foods and organic foods, and sale of ultra-processed products. The presence of ultra-processed products and related advertising is negatively scored; and items related to healthy eating and advertising are positively scored. In total, the instrument ranges from 0 to 15 points and allows for comparison between outlets.^15^

Data on average household income for each administrative region were obtained from the Census (2010) of the Institute for Research and Urban Planning of Curitiba (*Instituto de Pesquisa e Planejamento Urbano de Curitiba* – IPPUC).

The students’ questionnaires were precoded and double-entered into an electronic spreadsheet to correct possible inconsistencies. Data on availability and price of fruits and vegetables, as well as the quality index of the street markets, were entered into an electronic spreadsheet and the analyses were performed using the Stata 12.0 program (StataCorp, Texas, United States).

Descriptive analyses were carried out by calculating absolute (n) and relative (%) frequencies. The sample weights and the effect of the study design (survey) were considered in the analyses. Spearman’s correlation was used to assess the relationship between fruit and vegetable consumption and food availability and price. In order to correct the variation in food prices, their ratio was calculated by dividing the prices recorded in the data collection by the market prices of the Paraná Supply Center (*Central de Abastecimento* – CEASA), Curitiba unit. Therefore, the average of the price ratio of all fruits and vegetables was calculated according to the region.

The delimitation of the regions followed the IPPUC databases. With the address of the public outlets, georeferencing and the creation of thematic maps in QGIS 2.18 Las Palmas were carried out. Buffers were applied considering the area of influence in the school environment of 1.6 km and using the MMGIS complement (http://michaelminn.com/linux/mmqgis/) in QGIS 3.4. Subsequently, with the selection layer, the information was joined to obtain the public outlets belonging to each coverage radius of the school.

The choice of the area of influence was considered a possible distance to be covered to purchase food.^17–19^ The Moran’s local index (Local Indicator of Spatial Association – LISA) was applied with the SAGA plugin of the QGIS software, and the Geoda software was used. LISA employed the nearest-neighbor Ripley’s K function in the analysis.

The informed consent form was sent to those responsible for the students and handed out to students aged between 12 and 18 years. It was also handed out to those responsible for the stands of the public outlets. The research was approved by the Human Research Ethics Committee of the Health Sciences Sector (CEP/SD) of Universidade Federal do Paraná (UFPR) (protocol No. 1426615/2016).

## RESULTS

A total of 1,623 students were selected, of which 1,232 participated in the research (response rate of 75.9%), accounting for 51.2% boys and 90.9% students aged between 10 and 16 years. A total of 43.4% adolescents reported a regular intake of fruits, and 67.0%, of vegetables. Most of them (63.6%) reported living 20 minutes or less from school, and 39.4% stated they spent less than 10 minutes to walk to the school. Half of the students indicated that they bought food once a week or more in outlets near the school (52.2%).

The largest number of fruit varieties was identified in region 4 (p50=31.5). The highest price ratios of fruits were found in region 6 (3.06), being 2.7 times higher than the regions with the lowest price ratios: 3 (1.13) and 1 (1.14). The region with the greatest variety of vegetables was region 7 (35.0). The highest price ratios for vegetables were found in region 6 (3.64); and the lowest, in region 5 (1.84). No public outlet for the sale of fruits and vegetables was found in region 10 (Table 1).

**Table 1:** Frequency of regular consumption among students and median of verified varieties and the price ratio of fruits and vegetables in regions of Curitiba, Paraná, Brazil, 2018.

Region	Fruits (%)*	Vegetables (%)*	Fruits		Vegetables		HFSI	Average household income (BRL)
			Variety	Price (BRL)	Variety	Price (BRL)		
1	52.9	68.1	15.5 (14.0–17.0)	1.14 (1.12–1.16)	28.5 (28.0–29.0)	2.01 (2.00–2.03)	14 (3–15)	2,012.76
2	41.6	64.4	17.8 (8.0–28.0)	1.69 (1.38–2.19)	26.8 (17.0–34.0)	2.23 (1.98–2.52)	13 (11–15)	3,727.49
3	40.1	74.0	24.0 (21.0–32.0)	1.13 (1.13–2.99)	26.0 (21.0–39.0)	2.35 (1.89–2.83)	14 (3–15)	2,837.22
4	45.1	68.8	31.5 (27.0–34.5)	1.57 (1.20–2.32)	23.3 (7.0–35.0)	2.46 (1.95–2.93)	13 (11–14)	3,133.99
5	35.4	61.2	18.8 (18.0–18.0)	1.25 (1.25–1.25)	23.0 (23.0 - 23.0)	1.84 (1.84–1.84)	8 (3–13)	2,148.14
6	40.3	68.6	17.5 (8.8–26.3)	3.06 (2.55–4.32)	27.8 (17.0–36.0)	3.64 (3.02–5.35)	13 (12–13)	6,438.71
7	33.5	73.8	30.0 (8.5–30.0)	1.39 (1.36–1.98)	35.0 (12.5–35.0)	2.15 (1.98–3.00)	12.5 (7.5–14)	2,882.50
8	51.6	60.7	13.3 (10.0–27.0)	2.56 (2.33–4.35)	22.5 (14.0–40.0)	3.10 (2.62–5.98)	13 (11.5–13)	5,297.41
9	39.2	59.6	9.0 (2.0–31.0)	1.79 (1.44–2.51)	24.0 (19.0–30.0)	2.30 (2.07–3.10)	12.5 (11.5–13)	4,823.93
10	41.1	65.4	0§	0§	0§	0§	0§	1,674.84

* Estimates corrected for design effects and sample weights. Availability and price of fruits and vegetables and HFSI of the street markets: median (p50), 25^th^ percentile (p25), and 75^th^ percentile (p75) values

§ region 10 had no public outlet for the sale of fruits and vegetables; HFSI: Healthy Food Store Index; average household income – Demographic Census, 2010.

The prevalence of regular consumption of vegetables in the regions was correlated with their variety (r=0.82; p=0.007). Conversely, there was no correlation between the prevalence of regular fruit consumption and variety (r=-0.63; p=0.067). The average of vegetable prices showed a correlation with the average of fruit prices (r=0.87; p=0.003).

The median of HSFI was 13 points, with a more heterogeneous distribution in regions with lower income. However, no correlation was observed between the two variables (r=-0.13, p=0.74).

**Figure 1:**
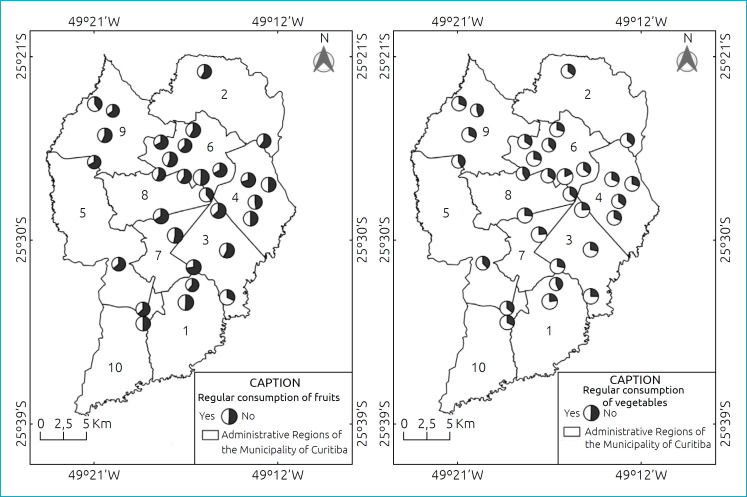
Regular consumption of fruits and vegetables in state schools in Curitiba, Paraná, Brazil, 2016 to 2017.

Only five schools had more than 50% of students with fruit consumption deemed regular, namely: school 2, region 1, with 68.0%; school 6, region 4, with 50.0%; school 11, region 4, with 52.0%; school 24, region 8, with 61.2%; and school 27, region 8, with 60.0%. Thus, regular fruit consumption ranged between 26.8% (school 21, region 7) and 68.0% (school 2, region 1). All schools had more than 50% of students with adequate consumption of vegetables, with frequencies ranging from 54.8% (school 29, region 9) to 82.2% (school 17, region 6) (Figure 1).

**Figure 2:**
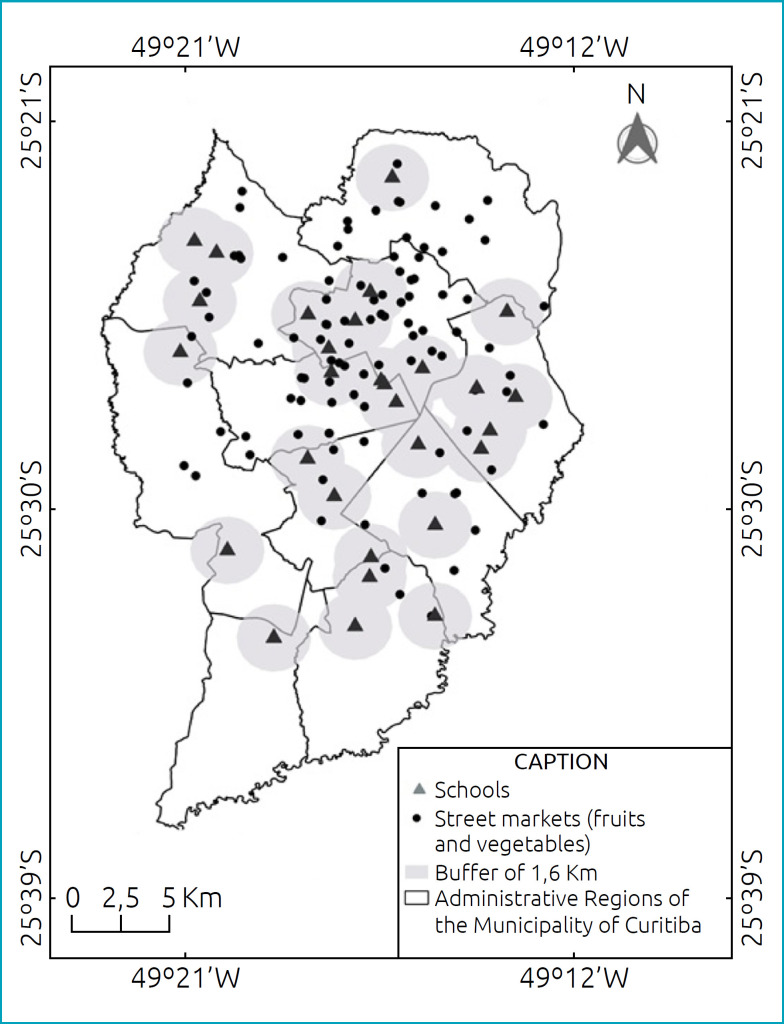
Distribution of schools and fruit and vegetable outlets in Curitiba, Paraná, Brazil, 2016 to 2018.

Schools in the northern and central regions of the city had greater availability of outlets. A total of 22 schools had public outlets with availability of fruits and vegetables in their vicinities, accounting for 72 possibilities of supply for these foods. Nevertheless, the same food outlet could be in the surroundings of up to three different schools — 46 public outlets with the supply of fruit and vegetables were located in the vicinity of the study schools. The greatest distance found between a school and a public outlet selling fruits and vegetables was of 5,780 m, with the school located in region 10 and the outlet in region 1. The shortest distance found was of 105 m, with the school and outlet located in region 4 (Figure 2).

**Figure 3:**
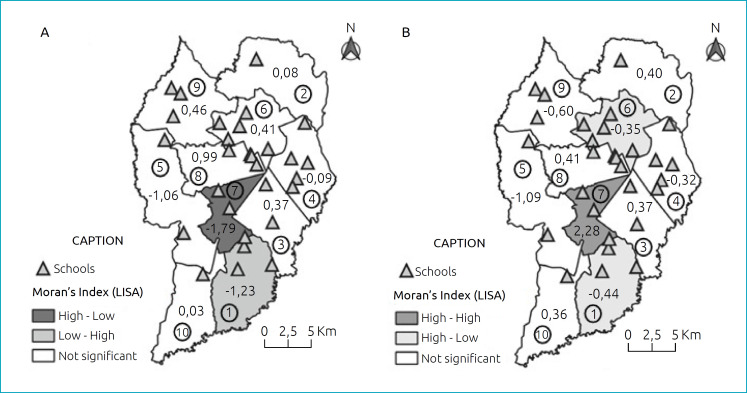
Moran’s Index between consumption and supply of fruits and vegetables among regions in the city of Curitiba, Paraná, Brazil, 2016 to 2018.

The Moran’s local index identified spatial autocorrelation of fruit consumption and supply in regions 1 and 7. In region 1, there was a low-high autocorrelation, that is, low fruit consumption for high supply (p=0.001); and, in region 7, high fruit consumption for low supply (p=0.001) (Figure 3). The Moran’s local index identified autocorrelation of vegetables in regions 1, 6, and 7, with high consumption and low supply in regions 1 and 6 (both p=0.001) and high consumption and high supply in region 7 (p=0.05) (Figure 3).

## DISCUSSION

The regular consumption of fruits and vegetables reported by adolescents from Curitiba was 43.4 and 67.0%, respectively. In the city of Pelotas, state of Rio Grande do Sul, Brazil, a study on elementary school students observed that the prevalence of regular consumption of fruits was 42.1%, similar to those found in the present study; concerning vegetables, it was 20.2%.^20^ In the northeast region, in the municipality of Caruaru, state of Pernambuco, Brazil, 17% of students ate fruits four to six times a week, and 8.9% ate vegetables at the same frequency.^21^ In Curitiba, there are differences in the supply of fruits and vegetables in public outlets in the vicinity of the school environment. This supply greatly varies for schools located in the most central regions of the city, and it is null in other school surroundings. When verifying the distribution of food markets in the city of Jundiaí, state of São Paulo, Brazil (2017), it was observed that outlets, such as street markets and fruit and vegetable markets, were concentrated in more central regions of the city.^22^

In the present study, the fruit and vegetable varieties showed differences between the regions, and there was no supply of these foods in one administrative region. The difference between the region with the highest median of fruit varieties was 3.5 times that with the lowest median; for vegetables, this variation was smaller (1.56 times). Difference in prices between the regions was high, being almost three times greater than in other regions. Moreover, according to the Moran’s index, there was spatial correlation between consumption and supply, with high supply and high consumption only in one region and in relation to vegetables.

Among adolescents from the city of São Paulo, state of São Paulo, Brazil, it was found that the presence of street markets closer to the residence (500 m) was associated with greater consumption of fruits and vegetables. Higher consumption was positively associated with higher household income.^6^ Most adolescents from Curitiba reported living near the school. Among the 30 schools evaluated, 22 were supplied with fruit and vegetables by public outlets in their vicinities, with a radius similar to that of the aforementioned study.

In Curitiba, regions with lower income had a lower supply of stands (in street markets) selling products at a fixed price, subsidized by the city hall.^23^ A 1% reduction in the price of fruits and vegetables would result in an increase of 0.79% in the total caloric share purchased by families.^24^ These studies demonstrate the importance of reducing the prices of such foods as a way of encouraging consumption, considering that people with lower purchasing power spend large part of their income on food and would require greater subsidies to achieve an adequate nutrition.^25^

Most regions had high median values for the classification of the HFSI index, with greater heterogeneity in the distribution among low-income regions. Nevertheless, the lack of correlation between the two variables indicates a certain homogeneity of the index in the regions. Conversely, there is a spatial difference in the amount of such outlets in the municipality’s regions, with greater concentration in the higher-income regions and in the more central ones.^23^

Although this study did not assess the income of adolescents, there was variation in the food price ratio, which demonstrates that the population can spend a higher percentage of their income depending on the region in which they reside. However, the highest prices were observed in regions with higher average income in the school environment, indicating the socioeconomic differences between regions.

In Paraná, the Department of Education recommends enrolling students in schools near their homes.^26^ Thus, it can be inferred that there is a partial overlapping of the school environment with the residential environment for most students. Furthermore, these outlets can be used by families to purchase fruits, vegetables and other products. Most families report less than 10 minutes to go from their houses to the place where they usually buy foods. The availability of fruit and vegetable stores within a radius of up to 100 m from the residence is a predictor of vegetable consumption, which highlights the importance of small neighborhood markets in promoting consumption.^27^

It is worth noting that street markets, due to opening hours, prices, and product characteristics, may not be the preferred spaces for the purchase of fruits and vegetables by adolescents, who can spend their resources on snacks, fast food and sweets.^28^ In this group, there is a dualistic view of food, in which healthy foods are associated with the family, and fast food is associated with pleasure and socializing with friends.^29^

The limitations of this study include that only public outlets selling fruits and vegetables were evaluated, disregarding the private markets of foods – such as supermarkets and fruit and vegetable markets. Information regarding the students’ addresses were not available, but most indicated that they lived less than 20 minutes from the school, and a significant portion reported shopping for food in its vicinity. In addition, the study was not designed to be representative of each school. In each one, students from one class or more were drawn to participate. Considering sample weights and the effect of the study design on the analyses partially corrects these aspects. Furthermore, the drawing of schools guaranteed at least one school per administrative region, which expands the geographic distribution of students.

The findings of this study demonstrate the inequalities in the supply of fruit and vegetables from public outlets in the vicinity of state schools in the city, and also that most adolescents have adequate vegetable consumption, but low fruit consumption. Associations were observed between the supply of these foods and the adequacy of consumption by adolescents, but spatial autocorrelations indicated differences between the consumption and supply of such foods, reinforcing the importance of studying other characteristics of the food environment. Studies on the food environment support public policies for promoting healthy eating habits by the spatial and economic facilitation of these foods.
